# Management of Intermittent exotropia in childhood: current concepts of the literature and the experts

**DOI:** 10.5935/0004-2749.2022-1001

**Published:** 2025-02-11

**Authors:** Luisa Moreira Hopker, Julia Dutra Rossetto, Fabio Ejzenbaum, Luis Carlos Ferreira de Sá, Geraldo de Barros Ribeiro, Tomás Scalamandré Mendonça, Mauro Goldchmit, Iara Debert

**Affiliations:** 1 Ophthalmology Department, Hospital de Olhos do Paraná, Curitiba, PR, Brazil; 2 Pediatric Ophthalmology Department, Instituto de Puericultura e Pediatria Martagão Gesteira, Universidade Federal do Rio de Janeiro, Rio de Janeiro, RJ, Brazil; 3 Ophthalmology Department, Santa Casa de Misericórdia de São Paulo, São Paulo, SP, Brazil; 4 Ophthalmology Department, Instituto da Criança, Hospital das Clínicas, Faculdade de Medicina, Universidade de São Paulo, São Paulo, SP, Brazil; 5 Ophthalmology Department, Centro Universitário de Belo Horizonte, Belo Horizonte, MG, Brazil; 6 Ophthalmology and Visual Science Department, Escola Paulista de Medicina, Universidade Federal de São Paulo, São Paulo, SP, Brazil; 7 Ophthalmology Department, Hospital CEMA, Sao Paulo, SP, Brazil; 8 Instituto Strabos, Sao Paulo, SP, Brazil; 9 Ophthalmology Department, Hospital das Clínicas, Faculdade de Medicina, Universidade de São Paulo, São Paulo, SP, Brazil

Intermittent exotropia X(T) occurs in approximately 1% of the population aged <11 years^(1)^. Although extensively investigated, controversies surround its propaedeutics, treatment, and specialists^(2)^. The disease course has also not been clearly defined. Some studies have demonstrated the absence of change over time^(3)^, whereas others have shown deviation improvements^(3,4)^ or even decompensation^(4)^. Opinions varied regarding treatment, both in clinical management with the use of occlusion^(5)^ or over minus lens prescription^(6)^ and in surgical treatment concerning the best time to perform surgery^(7,8)^ and the type of the surgical technique^(9,10)^. This paper summarizes the best practices for evaluating and treating X(T) based on expert opinion and a literature review.

Eight pediatric ophthalmology and strabismus specialists were invited to discuss the main points of X(T) semiology and treatment. They had at least 10 years of experience after ophthalmology residency, master’s, or doctorate degrees and trained in Brazil, abroad, or both. They answered a questionnaire concerning their management and were asked about their opinions on hypothetical X(T) cases.

A literature review was performed in PubMed database, using the following terms: “intermittent exotropia,” “surgery,” “management,” “occlusion,” and “overminus lenses” to support the points in question. The opinions of experts and literature review were divided in propaedeutics, clinical, and surgical treatment.

## Propaedeutics for X(T)

As part of propaedeutics, questioning parents about the deviation frequency is considered essential. Therefore, it was categorized as either above or below 50% of the day. The authors considered the deviation frequency during the examination to classify it as compensated or not. Observing the presence and frequency of spontaneous decompensation and the ability to control deviation during anamnesis and examination were highlighted.

Most experts used occlusion 30-60 min before an examination to induce the largest deviation. Some measured near deviation using +3.00 lenses and the distance measurements with the patient fixing on a target or through a window (at least 6 m).

Assessing whether the deviation occurred spontaneously or only after the fusion interruption by occlusion is essential. Another critical factor was to observe whether the recovery of the deviation occurred immediately after the removal of the occlusion, slowly, or if there was no recovery at all.

In the literature, some clinical data could contribute to the assessment of X(T) and its prognosis:

- Deviation angle versus prognosis: No cutoff value was established for the deviation angle. This could be considered a worse prognosis because the deviation control contributes more to the prognosis than its magnitude. A large-angle deviation with reasonable control generally had a better prognosis in the development of binocular vision than a small angle deviation with poor control^(11)^.
- Deviation control: Although scales can stratify the ability of patients to control deviation, control was most often variable, making classification by well-defined scores difficult. Family observations were employed to complement the findings observed during the consultation, as it had adequate variability, mainly related to how much time the guardian spends with the child^(11)^.
- Despite its rarity, stereopsis deterioration was considered an inadequate prognostic factor, as the definition of X(T) assumes normal stereopsis. The measurement of distance stereopsis was infrequently used, but it could provide more data regarding the deterioration of binocular vision^(11)^.

## Clinical treatment of X(T)

Refractive errors, such as high hyperopia, low-to-moderate myopia, or anisometropia, should be corrected to improve visual acuity^(12)^ and stereoacuity, improving deviation control^(13)^. Myopia must be fully corrected, and hyperopia >4.00 D or anisometropia >1.50 D should be corrected without significant discounts, as these patients might have poor accommodation^(2,13)^. Low myopia and astigmatism should be prescribed, as they improve deviation control^(12)^.

All authors suggested using anti-suppressive occlusion until surgery with an occlusion use of 1-3h, usually in the dominant eye, alternating if there was no dominance in one eye. for treating X(T) with occlusion. A deviation frequency improvement during the use of the occlusion without changing the outcomes of surgery was reported. The occlusion performance was reported to make no difference in the deterioration of the deviation^(14)^.

Recently, better deviation control was reported after 3 and 6 months of alternating occlusion in children aged 3-8 years. No difference in stereoacuity between the groups was reported^(15)^.

Some authors prescribed over minus lenses for children younger than the ideal age for surgery and whose deviation, even with occlusion, was uncontrolled. In such cases, -3.00 lenses were prescribed or added to the prescription.

In patients with X(T), over minus lenses reduced the deviation angle and improved control^(6,12)^. Generally, they were used in children aged <5 years to reduce the deviation frequency until surgery. This occurred because of accommodation, increase in accommodative convergence, and consequent reduction in deviation^(6,13)^. Generally, -1.00 and -3.00 D were added to the child’s cycloplegic refraction. The X(T) control improvement did not persist after lens use was discontinued ^(12)^. There were controversies about whether this approach could induce the onset of myopia in children^(16)^, and an increase in the myopic shift was recently suggested^(12)^.

## Surgical treatment

For most experts, the frequency of deviation and the capacity to control it are primary indications for surgery. Surgery was indicated if the deviation occurred longer than orthotropy during the day, with poor control, and generally deviations >25-30 prism-diopter (PD).

Other considerations included assessing asthenopia due to excess fusional convergence. Some experts indicated surgery only for children age ≥4 years. Others operated on uncompensated or large deviations (40-45 PD) at any age.

X(T) has a unique feature considering strabismus in children, as most patients had a single binocular vision and adequate stereopsis. Generally, the criteria for indicating surgery were deterioration of fusional control and a large deviation angle. In the literature, the reduction or loss of stereoacuity was also essential, but this test might not be reliable depending on the child’s age. No well-established recommendations for using these surgical indication criteria were reported^(11)^. There were controversies about the best age for surgery. Complete visual development was expected at around age 7 years. Hence, an undesired overcorrection postoperatively (consecutive esotropia) at a young age could lead to suppression, followed by amblyopia and loss of stereopsis^(11)^. However, recent studies have shown that early surgery (age 3-5 years) had better surgical success rates after 3 years of follow-up (8) and better motor alignment results^(7)^.

Regarding surgery type, for deviations up to 25 PD, bilateral lateral rectus recession had an adequate success rate of 86%, with a low overcorrection rate of 3%^(17)^. Lateral rectus recession was also more suitable for deviations, such as excess divergence. Still, pseudo-excess divergence and the basic X(T) type could be treated with unilateral recession-resection^(11,18)^. In a recent metaanalysis, for the basic type, both procedures, bilateral rectus recession and recession-resection, had similar success and chance of under or overcorrection^(19,20)^.

X(T) is a common ocular disease experienced by specialist and non-specialist ophthalmologists and lacks consensus on its management. This paper shows the leading scientific evidence and expert opinions on the diagnosis, clinical, and surgical management of X(T). Prospective case-control and randomized clinical trials are needed to establish individual objective criteria for clinical management, the best age for surgery, and surgical strategy.
